# Spillover effects of the sports industry on regional economic development: an analysis based on the spatial durbin model

**DOI:** 10.3389/fspor.2026.1804002

**Published:** 2026-05-19

**Authors:** Yuan Ren, Xing Gao, Xinxin Zhang, Jin Dong

**Affiliations:** 1 School of Physical Education, Myongji University - Natural Science Campus, Yongin-si, Republic of Korea; 2 School of Physical Education, Shanxi University School of Physical Education, Taiyuan, China

**Keywords:** double fixed regression model, heterogeneity analysis, regional economic development, spatial durbin model, sports industry

## Abstract

**Objective:**

Against the backdrop of deepening regional economic integration and the rapid development of the sports industry in China, this study systematically investigates the impact of the sports industry on regional economic development. Specifically, it identifies the industry's direct effects and spatial spillover effects while analyzing regional heterogeneity in influence mechanisms, thus addressing an existing research gap related to spatial connectivity.

**Method:**

Based on panel data from 21 provincial-level regions in China spanning 2015–2024, this study employs fixed-effects panel models and spatial Durbin models to analyze the relationship between the sports industry and regional economic development, while testing the underlying mechanisms.

**Results:**

First, the development of the sports industry significantly promotes regional economic growth, and this conclusion remains robust across multiple model specifications and robustness tests. Second, the impact of the sports industry on regional economic development shows clear regional heterogeneity: it is significant in the eastern region but not statistically significant in the central and western regions; Third, the spatiotemporal evolution of the sports industry and regional economic development shows similar patterns, characterized by low-value clustering and high-value polarization in eastern regions. Fourth, regional economic development exhibits a significant positive spatial correlation. Fifth, results from the spatial Durbin model indicate that while the sports industry promotes local economic growth, it produces significant negative spatial spillover effects on neighboring regions, forming a pattern characterized as “local promotion and neighboring suppression.”.

**Discussion:**

Empirical findings confirm the endogenous driving role of the sports industry as an emerging service sector in regional economic growth; however, its spatial effects are not uniformly positive. Negative spatial spillover effects may arise from the concentration of resources and associated “siphoning effects” during sports industry development, as well as interregional competition over event hosting, industrial layout, and factor allocation. This finding indicates that, in promoting the high-quality development of the sports industry, greater emphasis should be placed on enhancing regional coordination mechanisms and cross-regional industrial specialization systems. Such an approach would enhance local economic benefits while offsetting negative impacts on surrounding areas, thereby fostering coordinated regional economic development.

## 1 Introduction

In recent years, as the global economic structure has increasingly shifted from traditional manufacturing toward service-oriented and high-value-added industries, the sports industry has gradually emerged as an important driver of economic growth and industrial structure optimization (1). The sports industry not only encompasses traditional sectors such as sports competitions and performances, fitness and leisure services, and sporting goods manufacturing, but also produces substantial industrial linkage and diffusion effects through close integration with related industries, including culture, tourism, and health (2). Against the backdrop of consumption upgrading and increasingly diversified urban functions, the sports industry plays an important role in promoting employment, expanding domestic demand, and enhancing regional competitiveness, and is gradually emerging as a new engine of regional economic development (3).

Theoretically, the contribution of the sports industry to regional economic development goes beyond direct output expansion and employment generation. These contributions are manifested through spillover effects arising from mechanisms such as industrial linkages, innovation diffusion, human capital aggregation, and enhanced urban vitality (4). On the one hand, by integrating with diverse sectors including culture, tourism, health, technology, and manufacturing, the sports industry forms a multifaceted industrial chain centered on sports, thereby optimizing regional economic structures and improving factor efficiency. On the other hand, sporting events, sports consumption markets, and the spatial layout of sports facilities often exhibit significant geographical spillover effects. These spillovers may transcend administrative boundaries, exhibiting features of regional linkage and spatial diffusion (5). Therefore, examining the spillover effects of the sports industry on regional economic development is essential to understanding its external value and the underlying mechanisms of coordinated regional development.

However, existing research has primarily focused on the direct impact of sports industry scale on economic growth and lacks systematic identification and empirical examination of its spatial spillover effects. Within the context of China's evolving regional economic landscape and the accelerated formation of urban clusters and metropolitan areas, economic activities exhibit strong characteristics of spatial proximity and regional interaction. Traditional studies that neglect spatial correlation may result in biased estimations (6). Furthermore, regional differences in the foundations of sports industry development, resource endowments, industrial structures, and policy environments may affect both the magnitude and mechanisms of spillover effects generated by the sports industry (7). Therefore, it is necessary to introduce a spatial dimension into the analytical framework and employ spatial econometric models to systematically investigate the spillover effects of the sports industry on regional economic development.

Accordingly, this paper makes the following contributions: First, it theoretically investigates the relationship between the sports industry and regional economic development, thereby extending the existing literature. Second, it analyzes intercity differences in the sports industry and regional economic development from the perspective of regional heterogeneity and offers corresponding explanations. Third, by constructing a spatial econometric model, it addresses the limitations of conventional panel models that ignore spatial dependence.

## 2 Theoretical analysis and research hypotheses

### 2.1 Study on the direct impact of the sports industry on regional economic development

According to industrial economics and endogenous growth theory, the development of emerging industries acts as a key driver of high-quality regional economic growth. The sports industry's direct contribution to the local macroeconomy is mainly reflected in three dimensions: demand-pull effects, supply optimization, and agglomeration externalities (8).

From the demand-side perspective of the Keynesian multiplier, the expansion of consumption and investment directly drives economic growth (9). Centered on household sports consumption and public investment in sports infrastructure, the expansion of the sports industry effectively stimulates aggregate demand (10). Given the high income elasticity of sports consumption, improvements in living standards lead to non-linear growth in spending on sports services and products, thereby boosting regional output and economic activity through the multiplier effect.

From the supply-side perspective of endogenous growth, the sports sector, as a knowledge-, capital-, and service-intensive industry, is characterized by substantial human capital accumulation and technological progress (11). Its development not only attracts a high-quality workforce but also promotes the integration and application of advanced management practices and digital technologies (such as sports technology and wearable devices), thereby enhancing total factor productivity (TFP). The sports industry also exhibits strong industrial linkages. Through forward linkages (e.g., sports event broadcasting and sports tourism) and backward linkages (e.g., sports equipment manufacturing and venue construction), it promotes the coordinated expansion of upstream and downstream sectors, generating a strong synergy multiplier effect (12).

Based on Marshall's theory of industrial agglomeration, the concentration of the sports industry in a specific geographic area can generate economies of scale. Agglomeration reduces transaction costs and information asymmetry among firms and improves resource allocation efficiency through labor pooling, shared intermediate inputs, and knowledge spillovers (13). Based on this, the following hypothesis is proposed:

H1: Improvements in the level of sports industry development significantly promote regional economic growth.

### 2.2 Spatial spillover effects of the sports industry on regional economic development

According to Tobler's First Law of Geography and the theory of spatial econometrics, regional economic activities do not occur in isolation but exhibit strong spatial dependence. As a modern service industry characterized by openness and mobility, the sports industry's factors and industrial linkages often transcend traditional administrative boundaries, thereby generating spatial spillover effects in neighboring regions (14). According to Perroux's Growth Pole Theory and Myrdal's theory of circular cumulative causation, these spatial spillover effects are neither purely positive nor negative but reflect the interaction between spread effects and backwash effects (also known as polarization effects).

The sports industry may generate positive spatial spillover effects through spread effects. When the sports industry in a central region reaches a certain level of maturity, it promotes development in surrounding areas through industrial chain extension, technology diffusion, and demonstration effects. For example, large-scale sporting events in core cities (e.g., marathons and multi-sport games) generate substantial cross-regional visitor flows, directly promoting the development of transportation, catering, and tourism in neighboring regions (15). As land and labor costs rise in core regions, certain industrial segments, such as sports manufacturing, processing, and basic services, relocate to surrounding areas, thereby optimizing the industrial structure and improving economic efficiency in neighboring regions. This positive spatial autocorrelation provides a basis for the coordinated development of neighboring regions (16).

The sports industry may also generate negative spatial spillover effects through the backwash effect (siphoning effect). In the early stages of development or under conditions of imbalance, core regions become growth poles due to their superior infrastructure, policy support, and market potential (17). This strong centripetal force leads to a one-way flow of high-quality talent, capital, and premium event resources from surrounding areas to the core region (18). Peripheral regions, due to the outflow of production factors, experience pronounced shadow effects, thereby inhibiting economic growth in neighboring areas.

In summary, the spatial impact of the sports industry depends on the relative strength of spread and backwash effects. From the perspective of spatial competition and cooperation, this paper proposes the following two competing hypotheses for empirical testing:

H2a (Diffusion Effect Dominates): The development of the sports industry exhibits positive spatial spillover effects, meaning that its development in a given region can significantly promote economic growth in neighboring regions.

H2b (Backflow Effect Dominates): The development of the sports industry exhibits negative spatial spillover effects, meaning that its development in a given region significantly inhibits (or siphons) economic growth in neighboring regions.

## 3 Research design

### 3.1 Variable selection

#### 3.1.1 Core explanatory variable: sports industry (Si)

The dependent variable in this study is the sports industry (*Si*). The sports industry constitutes a comprehensive sector centered on sports-related activities, encompassing multiple domains and development stages. This study adopts the sports industry evaluation index system developed by Li Yanli (19) and measures SI across three dimensions: scale, structure, and efficiency. With respect to industrial scale, SI scale primarily reflects its overall volume and magnitude. Following Kang Lu (20) and Su Weizhou (21), the three dimensions are operationalized using 12 secondary indicators (see Table 1). Prior to model estimation, all indicators are normalized. To obtain relatively objective weights, we adopt Zhang Xinxin's method (22), combining the entropy weighting approach with a comprehensive development level evaluation model to measure SI. The detailed procedures are presented in Formulas (1–6).

**Table 1:** Evaluation system for the sports industry.

Primary indicator	Secondary indicator	Indicator description	Unit	Attribute	Weight
Industry Scale	Total Output of the Sports Industry	Total output value generated by sports industry production activities	100 million CNY	+	0.2043
	Value Added of the Sports Industry	Value added created by sports industry production activities	100 million CNY	+	0.0315
	Per Capita Sports Venue Area	Sports venue area per capita	m^2^/person	+	0.0711
	Number of Sports Venues	Total number of sports venues	10,000 units	+	0.0183
	Total Employment in the Sports Industry	Number of employees in urban units of the culture, sports, and entertainment sectors	10,000 units	+	0.0378
Industry Structure	Proportion of Sports Industry Output	Share of sports industry total output in GDP	%	+	0.2021
	Proportion of Sports Industry Value Added	Share of sports industry value added in GDP	%	+	0.1436
	Proportion of Fixed Asset Investment in Sports	Share of sports industry fixed asset investment in total social investment	%	+	0.0946
	Per Capita Sports Consumption Level	Share of per capita sports consumption in disposable income	%	+	0.0296
Industry Efficiency	Value-Added Rate of the Sports Industry	Ratio of sports industry value added to total sports industry output	%	+	0.1237
	Annual Growth Rate of Sports Industry Value Added	Increment of sports industry value added relative to the previous year	%	+	0.0256
	Revenue–Profit Margin of Sports Enterprises	Ratio of profits to revenues of sports enterprises	%	+	0.0179

Step 1: Normalize all the indicators using the following formula:

$$z_{i}=\{\begin{array}{c} \frac{x_{i}\text{-}b}{b\text{-}a}+0.001,\mathrm{for}\;\mathrm{positive}\;\mathrm{indicators},\\ \frac{b\text{-}x_{i}}{b\text{-}a}+0.001,\mathrm{for}\;\mathrm{positive}\;\mathrm{indicators}, \end{array}$$ (1)

where i(i=1,2,⋯,n) denotes the index of the indicators, *z_i_* denotes the normalized value of the *i* indicator, *x_i_* denotes the original value of the *i* indicator, *a* denotes the minimum value of all indicators, and *b* denotes the maximum value of all indicators.

Step 2: Calculate the ratio of the i-th indicator:

$$p_{i}=z_{i}/\sum_{i=1}^{n}z_{i}.$$ (2)

Step 3: Calculate the entropy of the i-th indicator:

$$e_{i}=-\frac{1}{\ln\;N}\sum_{i=1}^{n}\;p_{i}\ln\;p_{i},$$ (3)

where *N* represents the time span. According to the selected cross-sectional data of 2015, N=10.

Step 4: Calculate the variation coefficient of the i-th indicator:

$$g_{i}=1-e_{i}.$$ (4)

Step 5: Calculate the weight of the i-th indicator using the following formula:

$$w_{i}=g_{i}/\sum_{i=1}^{n}g_{i},$$ (5)

where *g_i_* is the weight of the j-th indicator.

Step 6: Calculate the comprehensive development index of the sports industry, which is expressed as:

$$u=\sum_{i=1}^{n}w_{i}z_{i},$$ (6)

where $\sum_{i=1}^{n}w_{i}=1$.

#### 3.1.2 Dependent variable: regional economic development (Red)

Following Zhao Jingxin et al. (23), we construct an index system to evaluate regional economic development based on four dimensions: economic development, innovation, coordination, and green development (Table 2). We employ the entropy method to measure regional economic development, and the specific calculation steps are described above.

**Table 2:** Evaluation system for regional economic development.

Mary dimension	Secondary indicator	Unit	Attribute	Weight
Economic Development	Regional GDP	100 million yuan	+	0.0213
	GDP Growth Rate	%	+	0.029
	Fixed Asset Investment Growth Rate	%	+	0.102
	Per Capita Fiscal Revenue	yuan	+	0.0722
Innovation Development	R&D Expenditure of Industrial Enterprises above Designated Size as a Share of GDP	%	+	0.0964
	Number of Invention Patents per 10,000 People	units	+	0.1668
	Number of High-tech Enterprises	units	+	0.0786
Coordinated Development	Per Capita Disposable Income	yuan	+	0.0992
	Per Capita Consumption Expenditure	yuan	+	0.1599
	Fiscal Revenue as a Share of GDP	%	+	0.0278
Green Development	Urban Sewage Treatment Rate	%	+	0.0221
	Harmless Treatment Rate of Domestic Waste	%	+	0.0722
	Energy Consumption per Unit of GDP	tons/10,000 yuan	−	0.0344
	Electricity Consumption per Unit of GDP	kWh/10,000 yuan	−	0.0177

#### 3.1.3 Control variables

To mitigate potential omitted variable bias and accurately isolate the net spatial and direct impacts of the sports industry on regional economic development, this study incorporates a set of control variables. Drawing upon regional economics and endogenous growth theories, these variables are selected based on their fundamental roles in shaping regional economic trajectories (24–26). The theoretical rationale and measurement for each control variable are detailed as follows:

##### 3.1.3.1 Infrastructure development (*Id*)

Measured by the total operational mileage of railways and highways within each region. A well-developed transportation infrastructure network is a fundamental prerequisite for regional economic growth. It significantly reduces spatial transaction and transportation costs, facilitates the cross-regional mobility of production factors (e.g., capital, labor, and technology), and strengthens market accessibility. Consequently, infrastructure development not only improves local resource allocation but also generates positive spatial spillover effects, thereby directly driving regional economic integration and expansion.

##### 3.1.3.2 Fixed asset investment (*Fai*)

Measured by the total actual investment in fixed assets across all sectors in each region. According to neoclassical economic growth models, physical capital accumulation is a primary engine of economic expansion. Fixed asset investment accelerates regional economic development through two channels: in the short term, it stimulates aggregate demand via investment multiplier effects; in the long term, it expands regional production capacity, facilitates industrial upgrading, and provides the necessary material foundation for sustained economic growth.

##### 3.1.3.3 Human resource level (*Hrl*)

Proxied by the proportion of workers in the cultural, sports, and entertainment sectors relative to the total employed population. Endogenous growth theory posits that human capital and specialized labor are critical drivers of sustained economic development. A higher concentration of human resources in these modern service sectors reflects a region's structural transition toward a knowledge- and service-driven economy. This concentration enhances overall labor productivity, fosters localized knowledge spillovers, and thereby accelerates regional economic modernization.

##### 3.1.3.4 Urbanization level (*Ul*)

The ratio of the urban permanent resident population to the regional permanent resident population. Urbanization is intrinsically linked to agglomeration economies and economies of scale. High urbanization rates facilitate the spatial concentration of consumer markets and production activities, which dramatically improves resource matching efficiency. Furthermore, urban environments provide the essential demographic and spatial carriers for the expansion of the tertiary sector, directly boosting regional economic vitality and consumer demand.

##### 3.1.3.5 Degree of government intervention (*Dgi*)

Measured by the ratio of general public fiscal budget expenditure to regional GDP. From a macroeconomic perspective, government fiscal policy plays a pivotal role in shaping regional economic development. Appropriate government intervention can effectively correct market failures, provide essential public goods (such as public infrastructure and sports facilities), and optimize industrial structures through targeted expenditures. Thus, the scale of local government spending captures the institutional and macroeconomic environment that significantly influences regional economic performance.

#### 3.1.4 Data sources

Due to the relatively late development of the sports industry in certain provinces and the incomplete availability of relevant data, many provincial-level administrative regions did not begin to release official statistics until 2015. To ensure data reliability, methodological rigor, and overall data quality, this study excludes these provinces and corresponding years from the analysis.Instead, in this study, the unit of analysis is the provincial-level administrative region. The dataset includes panel observations for 21 provinces or municipalities in China (namely Beijing, Tianjin, Hebei, Shanxi, Inner Mongolia, Liaoning, Shanghai, Jiangsu, Zhejiang, Anhui, Fujian, Jiangxi, Shandong, Henan, Hubei, Hunan, Guangdong, Chongqing, Sichuan, Guizhou, and Yunnan) over the period 2015–2024, resulting in 210 province–year observations.The research data were obtained from provincial sports bureaus, the National Bureau of Statistics, annual editions of the China Statistical Yearbook and the China Sports Yearbook, and the China National Research Data Service (CNRDS).

Because some indicators related to the sports industry are not consistently reported in certain years, a small proportion of missing values appears in the dataset. After preliminary screening, missing observations account for 3.27% of the total data points. To address this issue while preserving the temporal continuity of the panel dataset, linear interpolation was employed to estimate missing values within the time series of each province. This approach is widely adopted in panel data studies when missing values occur intermittently and when the underlying indicators exhibit relatively smooth temporal variation. Specifically, the interpolation procedure estimates missing observations using adjacent years' values within the same province, ensuring that the imputed values remain consistent with the local temporal trend. Since the proportion of missing data is relatively small, the interpolation treatment is unlikely to introduce systematic bias into the empirical results.

To ensure the robustness of the empirical results, descriptive statistics were rechecked after data interpolation to confirm that no abnormal values or structural distortions were introduced into the dataset.

### 3.2 Model setup

#### 3.2.1 Reference model

To test H1, this paper constructs Equation 7 to examine the impact of the sports industry (*Si*) on regional economic development (*Red*). If H1 holds, the regression coefficient (*α*1) for Si is expected to be significantly greater than 0, indicating that Si promotes regional economic development. The model also incorporates selected control variables: infrastructure development (*Id*), fixed asset investment (*Fai*), human resource level (*Hrl*), urbanization level (*Ul*), and degree of government intervention (*Dgi*). Additionally, *Year* represents a time fixed effect, *City* denotes a region fixed effect, and *ε* is the random error term. These components control for the common impact of unobservable temporal factors on sports industry development.

$$Red_{it}=\alpha_{0}+\alpha_{1}Si_{it}+\alpha_{2}Id_{it}+\alpha_{3}Fai_{it}+a_{4}Hrl_{it}+\alpha_{5}Ul_{it}+\alpha_{6}Dgi_{it}+Year_{t}+City_{i}+\epsilon$$ (7)

### 3.2 Spatial model

This paper constructs a spatial Durbin model to identify the spatial spillover effects of the sports industry on regional economic development, with the following specification (Equation 8):

$$Red_{it}=\beta_{0}+ΡWRed_{it}+\beta_{1}Si_{it}+\beta_{2}Control_{it}+\gamma_{1}WSi_{it}+\gamma_{2}WControl_{it}+Year_{t}+City_{i}+\epsilon_{it}$$ (8)

Among these, *Ρ* represents the spatial lag coefficient; ɤ1 and ɤ2 denote the spatial autocorrelation coefficients for sports industry and control variables, respectively, with other parameters defined as above. W is the spatial weight matrix, which in this study adopts a geographic adjacency matrix. Element values are primarily determined based on geographic proximity principles: when two regions are geographically adjacent, the corresponding matrix element is assigned a value of 1; otherwise, it is 0, thereby indicating the direct connection between them.

## 4 Empirical results analysis

### 4.1 Descriptive statistical analysis、correlation and multicollinearity tests

To better understand variation across variables and obtain deeper insights into sample trends, descriptive statistical analysis was initially performed for all variables. To mitigate potential heteroskedasticity in the empirical regression, regional economic development, infrastructure construction, and fixed asset investment were transformed using natural logarithms. Table 3 reports the descriptive statistics for the main variables in the model. The total number of observations in this study is 210. The results show that the dependent variable, “Regional Economic Development (*Red*),” has a mean of 0.1620 and a standard deviation of 0.1101. The core independent variable, “Sports Industry (*Si*),” has a mean of 0.1130 and a standard deviation of 0.0876, indicating that there is a certain degree of variation in the levels of the sports industry and economic development across the sample. Regarding the control variables, “Fixed Asset Investment” exhibits the highest mean (8.3267) and the greatest volatility (standard deviation of 0.7207), whereas the “Degree of Government Intervention” shows the smallest dispersion (standard deviation of 0.0097). Overall, the data distribution of all variables falls within a reasonable range with no obvious anomalies, providing a reliable data foundation for the subsequent empirical tests.

**Table 3:** Descriptive statistics analysis.

Variable name	Observations	Standard deviation	Minimum value	Maximum value	Mean
Sports Industry	210	0.0876	0.0236	0.5475	0.113
Regional Economic Development	210	0.1101	0.0183	0.4522	0.1620
Infrastructure Development	210	0.3993	4.1274	5.6424	5.1571
Fixed Asset Investment	210	0.7207	4.7296	11.3351	8.3267
Human Resource Level	210	0.3016	0.0037	1.337	0.2853
Degree of Government Intervention	210	0.0097	0.8713	0.9174	0.8951
Urbanization Level	210	0.1761	7.4364	8.2683	7.8560

Tables 4, 5 report the correlation coefficient matrix and the Variance Inflation Factor (VIF) test results among the variables, respectively. As shown in Table 4 (the lower left triangle presents Pearson correlation coefficients, while the upper right triangle presents Spearman rank correlation coefficients), the core independent variable “Sports Industry (Si)” and “Regional Economic Development (Red)” exhibit a significant positive correlation at the 1% level in both tests (Pearson r = 0.597; Spearman r = 0.519). This preliminarily suggests that the development of the sports industry has a positive driving effect on the regional economy. Although there are correlations among the main variables, the coefficients are generally low. To further examine the potential threat of multicollinearity in the model, the VI*F* test results in Table 5 indicate that the VIF values of all explanatory variables range from 1.06 to 4.71. The maximum value of 4.71 is strictly below the widely accepted academic threshold of 5, with a mean VIF of only 2.11. In conclusion, these results demonstrate that there are no severe multicollinearity issues within the variable system of this study, making it suitable for subsequent regression analysis.

**Table 4:** Correlation analysis.

Variable	Si	Red	Id	Fai	Hrl	Dgi	Ul
Si	1	0.519***	−0.043	0.086	0.234***	0.168**	0.083
Red	0.597***	1	0.209***	0.127*	0.318***	0.455***	0.418***
Id	−0.015	0.411***	1	0.146**	0.234***	0.390***	0.485***
Fai	−0.071	0.028	0.003	1	−0.122*	0.314***	−0.017
Hrl	0.179***	0.361***	0.561***	0.188***	1	0.183***	0.417***
Dgi	0.280***	0.458***	0.291***	0.029	0.015	1	0.121*
Ul	0.139**	0.508***	0.475***	−0.023	0.505***	0.083	1

Lower-triangular cells report Pearson's correlation coefficients, upper-triangular cells are Spearman's rank correlation.*** *p* < 0.01, ** *p* < 0.05, * *p* < 0.1.

**Table 5:** VIF inspection.

Variable	VIF	1/VIF
*Si*	2.15	0.4657
*Id*	4.71	0.2123
*Fai*	1.06	0.9396
*Hrl*	1.63	0.6123
*Dgi*	2.39	0.4180
*Ul*	3.39	0.2952
Mean VIF	2.11	

### 4.2 Benchmark regression analysis

To control for regional and time fixed effects, this study uses regional economic development as the dependent variable and the sports industry as the key explanatory variable. Infrastructure development, fixed asset investment, human resource levels, urbanization rates, and government intervention levels serve as control variables. A two-way fixed effects panel model was employed for estimation (Equation 7), and the results are reported in Table 6. Model *Red*(1) reports the baseline regression excluding control variables, whereas Model *Red*(2) includes control variables but does not incorporate regional or time fixed effects. Model *Red*(3) reports the benchmark specification, including control variables as well as both regional and time fixed effects. Across these three models, the regression coefficients for the key explanatory variable, the sports industry (*Si*), are 0.3223, 0.6959, and 0.2865, respectively. All coefficients are positive and statistically significant at the 1% level. These results indicate that the sports industry exerts a significant and positive effect on regional economic development, regardless of whether control variables are included.

**Table 6:** Results of benchmark regression analysis.

Variables	Red (1)	Red(2)	Red(3)
Si	0.3223***	0.6959***	0.2865***
	(4.66)	(6.63)	(4.10)
Id		0.1293***	0.1170**
		(4.77)	(2.15)
Fai		−0.0074	0.014
		(-1.16)	(0.30)
Hrl		0.0383**	0.0278*
		(2.05)	(1.40)
Dgi		−0.1504***	−0.1405***
		(−3.87)	(−2.87)
Ul		0.0553*	0.2825*
		(1.70)	(1.90)
year	YES	NO	YES
city	YES	NO	YES
R2	0.8020	0.4735	0.8145
F	20.73***	8.22***	5.71***
N	210	210	210

The values in parentheses represent the t-statistic. ***, **, and * denote significance levels of 1%, 5%, and 10%, respectively. The same applies below.

Thus, compared with traditional industries, the sports industry simultaneously stimulates consumption, attracts investment, and facilitates the diffusion of innovation, thereby generating multidimensional effects on regional economic growth. First, from an industrial economics perspective, the sports industry enhances the integrity and coordination of regional industrial systems by extending value chains and strengthening inter-industry linkages (12). The synergistic development of sports event operations, sports equipment manufacturing, sports services, and related cultural tourism activities facilitates more efficient resource allocation across sectors, thereby improving overall regional productivity. Second, from the perspective of economic growth theory, the development of the sports industry helps shift regional economies away from single-factor-driven growth and toward endogenous growth trajectories (2). On the one hand, the development of the sports industry relies heavily on human capital, brand equity, and the institutional environment. Its expansion inherently involves technological adoption, organizational innovation, and improvements in managerial efficiency, thereby fostering continuous knowledge accumulation and regional technology diffusion. On the other hand, by improving public service provision and urban livability, the sports industry enhances a region's attractiveness to high-quality labor and capital, thereby strengthening factor agglomeration and economies of scale (27). Furthermore, the sports industry indirectly enhances regional economic dynamism by shaping regional image, strengthening social capital, reducing transaction costs, and improving the business environment. Collectively, these mechanisms suggest that the sports industry is not merely an “outcome variable” of economic growth, but rather an increasingly important endogenous driver of high-quality regional development.

In terms of control variables, as shown in Models *Red (2)* and *Red (3)*, the regression coefficients for infrastructure development are 0.1293 and 0.1170, respectively. Both are significantly positive, indicating that improvements in infrastructure development stimulate regional economic growth. This may be because infrastructure, as the physical foundation for reducing geographical barriers, lowers transaction and transportation costs, thereby promoting the cross-regional flow and aggregation of production factors such as capital and labor. This provides a sustained endogenous force for expanding market scale and promoting high-quality regional economic development.

The regression coefficients for human resource levels are significantly positive in both models, indicating that improvements in human resources promote regional economic development. This may be because higher human resource levels enhance labor productivity and innovation capacity, thereby promoting the growth of the sports industry and regional economic development (28).

The regression coefficient for government intervention shows a significant negative effect, suggesting that excessive intervention may create a crowding-out effect on private investment and lead to resource misallocation and distorted market signals. This suppresses innovation at the micro level and reduces the efficiency of market-based resource allocation, thereby weakening endogenous growth momentum (29).

The regression coefficient for urbanization is significantly positive. This may be because urbanization promotes the spatial agglomeration of population and industries, generates economies of scale and agglomeration externalities, improves resource matching efficiency, and expands the consumer market, thereby supporting industrial upgrading and sustained regional economic growth.

The regression coefficient for fixed-asset investment is negative but not significant without fixed effects, and becomes positive but remains insignificant when both year and province fixed effects are included. This may be because, without fixed effects, omitted variables related to regional endowments and macroeconomic fluctuations bias the results, leading to a spurious negative relationship (30). After introducing two-way fixed effects to control for spatiotemporal heterogeneity, the positive role of capital accumulation is restored, but its marginal effect on high-quality output is weakened by real-world constraints, such as diminishing returns, investment misallocation, and overcapacity. As a result, this effect remains statistically insignificant.

### 4.3 Robustness tests

1. Truncation Treatment

To further assess the robustness of the benchmark regression results and reduce the influence of extreme outliers on the estimated relationship between the sports industry and regional economic development, this study winsorized the core variables at the 1st and 99th percentiles and re-estimated the model. After replacing the original variables with the winsorized series and re-estimating the model, the results in Table 7 (*Red* 4) show that the coefficient on the sports industry remains positive and statistically significant at the 1% level. This result supports the robustness and reliability of the main findings.

2. One-period lag processing

**Table 7:** Robustness test results.

Variables	Red(4)	Red(5)	Red(6)	Low(1)
*Si*	0.2764***		0.4569***	0.1725***
	(4.05)		(5.76)	(3.64)
*L.Si*		0.3297***		
		(3.34)		
*Id*	0.1301***	0.1062**	0.0371	0.0480
	（3.65）	(2.26)	(0.59)	(0.38)
*Fai*	0.0174***	0.0154**	0.0087	0.0414***
	（2.74）	(2.27)	(1.42)	(3.34)
*Hrl*	0.0082	−0.0101	0.0093	−0.0870**
	（0.40）	(−0.44)	(0.37)	(−2.04)
*Dgi*	−0.0008	−0.0509	−0.0306	−0.0982***
	（−0.11）	(−0.37)	(−0.17)	(−3.38)
*Ul*	0.3163**	0.6693***	0.3612*	0.7499***
	（2.09）	(3.31)	(1.93)	(4.07)
*year*	YES	YES	YES	YES
*city*	YES	YES	YES	YES
*R2*	0.8186	0.8063	0.8496	0.9107
*F*	6.24***	4.89***	8.46***	5.80***
*N*	210	189	170	210

As an additional robustness check, this study introduced a one-period lag（*L.Si*） of the core explanatory variable to examine potential lagged effects and to mitigate endogeneity concerns arising from reverse causality and contemporaneous shocks. As reported in Table 7 (*Red* 5), the coefficient on the lagged sports industry variable is 0.3297 and remains statistically significant at the 1% level. This finding indicates that the main conclusions remain valid after accounting for lagged effects, further confirming the robustness of the empirical results.

3. Exclude sample data from municipalities directly under the central government

Given the substantial differences between municipalities and other provinces in terms of economic structure, policy environment, and data characteristics, these observations may exert disproportionate influence or introduce bias into the regression estimates. Therefore, Beijing, Tianjin, Shanghai, and Chongqing were excluded from the sample, and the regressions were re-estimated. As reported in Table 7 (*Red* 6), the effect of the sports industry on regional economic development remains significantly positive under the benchmark specification, further supporting the robustness of the conclusions.

4. Substitution of the Dependent Variable

To avoid potential bias caused by the measurement method, this study follows Ren Bo (31) and replaces regional economic development with the level of openness (*Low*). This variable is measured as the ratio of total imports and exports to gross regional product and is then included in the regression model (*Low* 1). The robustness test results show that the impact of the sports industry on regional openness remains significant and consistent with the baseline results, indicating that the findings are robust to the substitution of the dependent variable.

### 4.4 Heterogeneity analysis

There are significant differences among China's regions in terms of natural conditions and levels of economic development. Based on the regional classification standards of the National Bureau of Statistics, the sample regions are divided into two major groups: the eastern region and the central and western regions. The eastern region includes Beijing, Tianjin, Hebei, Liaoning, Shanghai, Jiangsu, Zhejiang, Fujian, Shandong, and Guangdong; the central and western regions include Shanxi, Inner Mongolia, Anhui, Jiangxi, Henan, Hubei, Hunan, Chongqing, Sichuan, Guizhou, and Yunnan. Building on this, we further analyze the heterogeneous impact of the sports industry on regional economic development.

The results in Table 8 show that the impact of the sports industry on regional economic development exhibits clear regional heterogeneity. According to the regression results, the sports industry in the eastern region has a significant positive effect, whereas in the central and western regions, the effect is positive but not statistically significant. This may be attributed to the higher level of economic development and the more mature consumer market in the eastern region. On the one hand, residents in the eastern region have higher incomes, and their consumption structure has shifted from subsistence to development and enjoyment, with expanding demand for sports consumption, providing a solid market foundation for the sports industry. On the other hand, the region has a well-developed industrial system, allowing the sports industry to integrate with related sectors such as culture, tourism, exhibitions, the internet, and health services, forming a multi-sectoral synergy that amplifies its economic spillover effects (32). Furthermore, the eastern region has a high concentration of capital, technology, and talent. Combined with strong infrastructure and a favorable business environment, these factors promote the clustering of sports enterprises and the introduction of major sporting events, further strengthening its role in regional economic growth.

**Table 8:** Results of heterogeneity analysis.

Variables	Red(7)	Red(8)
	Eastern	Central
*Si*	0.3088*	0.2537
	（1.88）	(1.18)
control	YES	YES
year	YES	YES
City	YES	YES
R^2^	0.8374	0.6639
F	5.03***	3.08***
N	100	110

The values in parentheses represent the t-statistic. ***, **, and * denote significance levels of 1%, 5%, and 10%, respectively. The same applies below.

In contrast, although the sports industry in the central and western regions has a positive impact, this effect is not statistically significant due to several factors. First, the overall economic level in these regions is lower, with relatively low disposable income; sports consumption remains at an early stage, and market demand has not yet reached economies of scale. Second, the foundation of the sports industry is weak, with an incomplete industrial chain. The underdevelopment of sports services, event operations, and related industries limits value-added and spillover effects (33). Third, these regions experience an outflow of capital, talent, and management expertise. The lack of leading firms and major events hinders industrial agglomeration and economies of scale (34). In addition, in some regions, the sports industry remains government-led and focused on infrastructure, with underdeveloped market mechanisms and a time lag in converting inputs into economic benefits.

These regional disparities reflect the characteristics of China's economic spatial structure and industrial division of labor. The eastern region has entered a service-oriented development phase. As a key component of modern services, the sports industry is better integrated into industrial upgrading and growth. In contrast, the central and western regions remain in stages of industrialization and infrastructure improvement. Their growth relies on traditional industries and investment, and the sports industry accounts for a small share of the economy, making its short-term contribution limited.

### 4.5 Analysis of spatial spillover effects

#### 4.5.1 Spatiotemporal evolution of the sports industry and regional economic development

**Figure 1:**
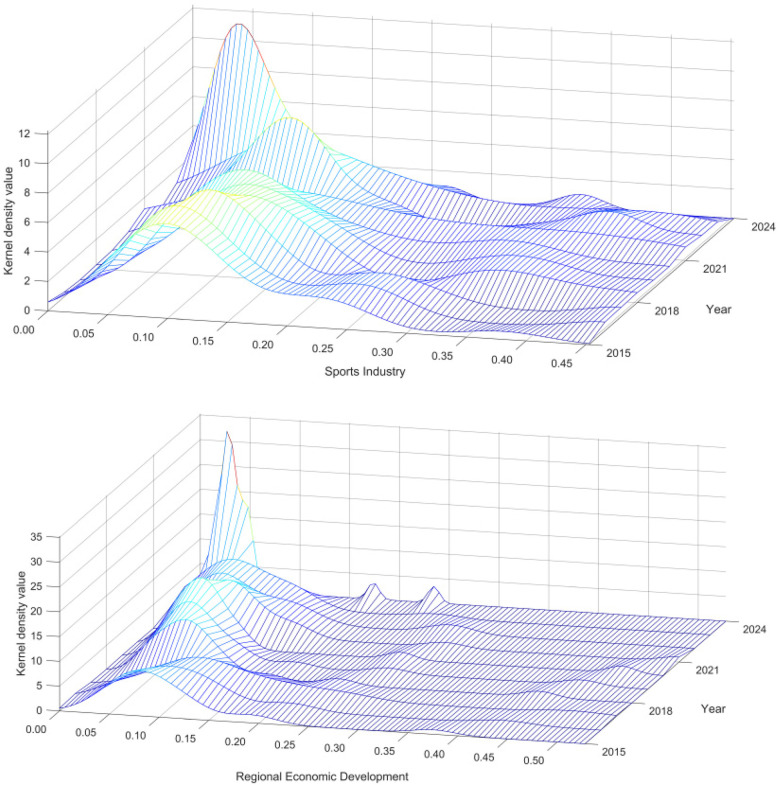
Heat Map of the sports industry and regional economic development.

**Figure 2:**
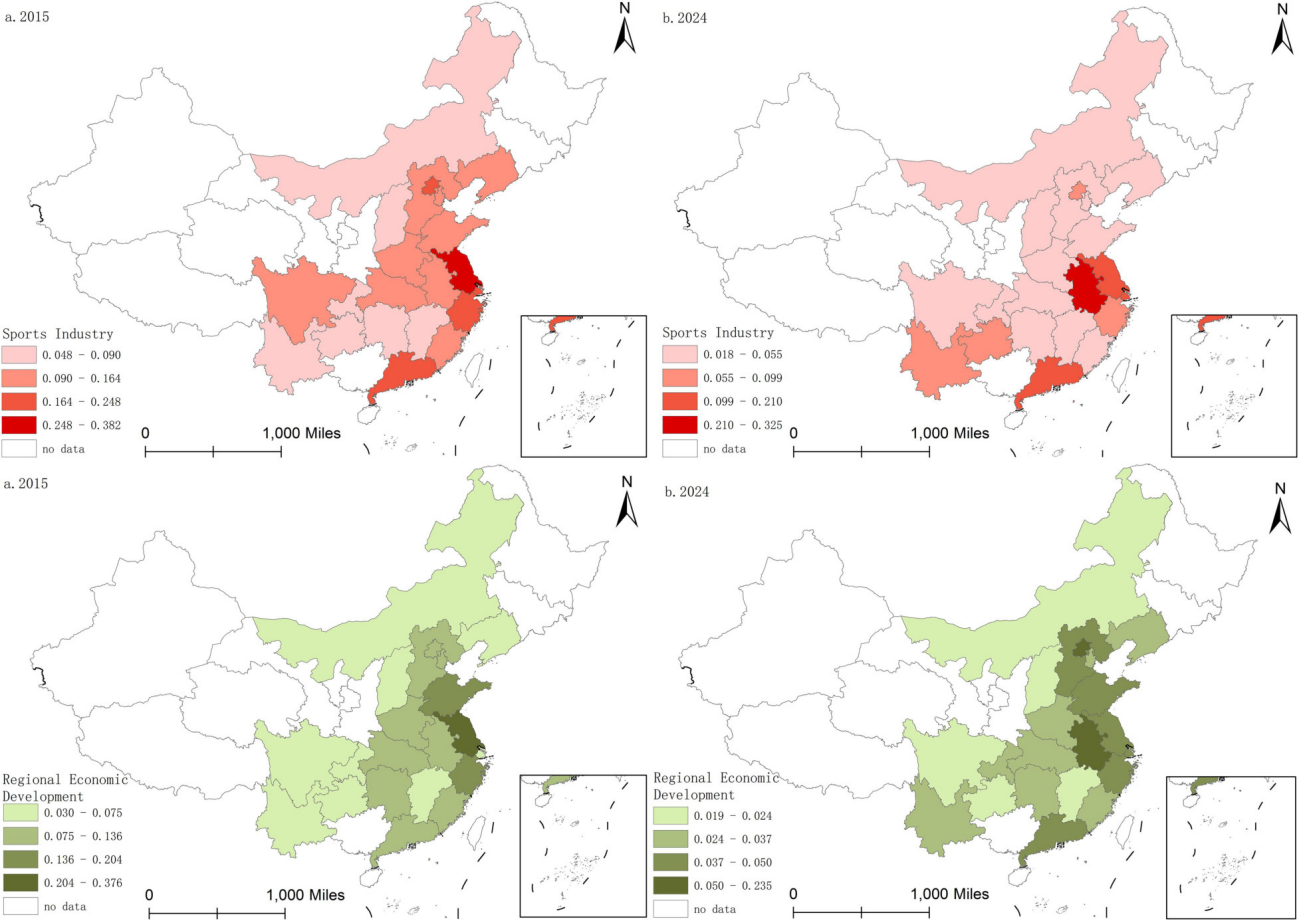
Spatio-temporal and evolutionary characteristics of the sports industry and regional economic development.

To characterize the spatiotemporal evolution of the sports industry and regional economic development across China’s provinces from 2015 to 2024, this study uses MATLAB and ArcGIS to visualize the data through kernel density estimation and the natural breaks method (35). The results are presented in Figures 1, 2.

To examine the dynamic evolution of the sports industry and regional economic development during the study period, this study employs three-dimensional kernel density estimation (KDE) to characterize temporal changes in the distribution, location, and dispersion of these two systems from 2015 to 2024. Overall, the kernel density distributions of both systems exhibit a clear right-skewed pattern with a single dominant peak, indicating significant regional imbalances.

Specifically, the main peak of the sports industry remains in the low-value range, with the peak rising over time and becoming narrower, indicating an intensifying low-level convergence effect. At the same time, the long right tail persists, suggesting that a few developed regions maintain a clear lead, and regional inequality remains pronounced. The evolution of regional economic development follows a similar pattern: the main peak also rises in the low-value range, and the long right tail is sometimes accompanied by a weak secondary peak, confirming spatial polarization and the divide between core and peripheral regions.

In summary, the two systems show consistent temporal evolution, characterized by low-value clustering and high-value polarization. This right-skewed clustering pattern reflects a potential siphon effect in core regions and provides empirical support for applying the Spatial Durbin Model (SDM) to examine spatial spillover effects of the sports industry.

As shown in the spatial distribution maps, from 2015 to 2024, both China's sports industry and regional economic development exhibit clear spatial heterogeneity and agglomeration, with a stable east–west gradient (higher in the east and lower in the west). Specifically, high-value areas of the sports industry are concentrated in eastern coastal regions (e.g., Jiangsu, Zhejiang, and Guangdong). During this period, the core–periphery pattern became more pronounced, while large areas of central, western, and northeastern China remained in contiguous low-value zones. Regional economic development shows a similarly stable pattern with clear spatial clustering.

In summary, a comparison of the two maps shows that high-value clusters of the sports industry and high-level regions of economic development largely overlap geographically. This spatial overlap indicates a close co-evolutionary relationship between the two systems. This non-random distribution and strong spatial dependence provide a basis for applying the Spatial Durbin Model (SDM) to examine spatial effects.

#### 4.5.2 Spatial correlation analysis

Before conducting spatial regression analysis, it is necessary to test whether spatial correlation exists in regional economic development. Therefore, this study selects the period from 2015 to 2024 and uses 210 provincial-level regions as the research sample. The new Moran's *I* index for regional economic development was calculated, and the results are reported in Table 9. The findings show that Moran's *I* is significantly positive at the 10% significance level, indicating that *Red* exhibits significant positive spatial autocorrelation. This suggests a clear spatial agglomeration effect in regional economic development. In other words, from the perspective of spatial distribution, neighboring regions tend to display similar levels of economic development, reflecting a clustered development pattern.

**Table 9:** Moran's Index for regional economic development.

Year	Moran's I	E(I)	Sd(I)	Z	*P*-value
2015	0.1893	−0.0500	0.1219	1.9622	0.05
2016	0.1668	−0.0500	0.1207	1.7956	0.07
2017	0.1610	−0.0500	0.1234	1.7094	0.08
2018	0.1818	−0.0500	0.1081	2.1444	0.03
2019	0.1408	−0.0500	0.1121	1.7019	0.08
2020	0.1768	−0.0500	0.1017	2.2309	0.03
2021	0.1612	−0.0500	0.1080	1.9549	0.05
2022	0.2754	−0.0500	0.1374	2.3692	0.01
2023	0.2488	−0.0500	0.1213	2.1391	0.01
2024	0.2001	−0.0500	0.1347	1.9137	0.02

#### 4.5.3 Spatial durbin model regression results

This study sequentially conducted the LM test, LR test, and Wald test (Table 10). All three tests reject the null hypothesis at the 1% significance level, indicating that the spatial Durbin model (SDM) is the most appropriate specification. Table 11 reports the estimation results of the SDM. The regression results show that the spatial autoregressive coefficient (*ρ*) of the spatial lag term is significantly positive at the 1% level, confirming that regional economic development exhibits significant spatial spillover effects.

**Table 10:** Results of LM,LR and wald tests.

Inspection method		Statistic	p
LM test	LM-error	12.57***	0.000
	Roubust LM-error	4.60***	0.003
	LM-lag	24.26***	0.000
	Robust LM-lag	16.29***	0.000
LR test	LR-SDM-SAR	19.27***	0.004
	LR-SDM-SEM	19.73***	0.003
WALD test	Wald_sdm_sar	20.14***	0.003
	Wald_sdm_sem	20.73***	0.002

*** indicates significance at the 1% level. The LM 、LR and Wald tests follow a chi-square distribution with 6 degrees of freedom (df = 6).

**Table 11:** Regression results for the spatial dubin model.

	(1)	(2)	(3)	(4)	(5)	(6)	(7)
Variables	Main	Wx	Spatial	Variance	LR_Direct	LR_Indirect	LR_Total
Si	0.2832***	−0.5844***			0.2781***	−0.5462***	0.8242***
	(4.53)	(3.47)			(4.35)	(3.15)	(4.47)
id	0.0596	−0.0124			0.0573	−0.022	0.0361
	(1.08)	(−0.10)			(1.08)	(−0.18)	(0.27)
fai	0.0203***	0.0365			0.0199***	0.0362	0.0562**
	(3.57)	(1.23)			(0.3.70)	(1.36)	(2.01)
hrl	0.0146	−0.0342			0.0149	−0.0292	−0.0142
	(0.054)	(−0.71)			(0.59)	(−0.65)	(−0.25)
dgi	0.0805	0.4972			0.0737	0.4822	0.555
	(0.70)	(1.28)			(0.66)	(2.32)	(0.409)
ul	0.3343**	0.9300**			0.3319**	0.9078**	1.2392***
	(2.29)	(2.30)			(2.23)	(2.15)	(1.36)
*ρ*			0.0571**				
			(3.06)				
sigma2_e				0.0012***			
				(7.69)			
Observations	210	210	210	210	210	210	210
R-squared	0.2583	0.2583	0.2583	0.2583	0.2583	0.2583	0.2583
Number of id	21	21	21	21	21	21	21
City	YES	YES	YES	YES	YES	YES	YES
Year	YES	YES	YES	YES	YES	YES	YES

The values in parentheses represent the t-statistic. ***, **, and * denote significance levels of 1%, 5%, and 10%, respectively. The same applies below.

With respect to the spillover effects of the sports industry, the estimated spatial interaction coefficient is −0.5844 and is statistically significant. This suggests that while the sports industry promotes economic development within a region, it generates a negative spillover effect on neighboring regions, thereby inhibiting their economic development.

Further decomposition of spatial spillover effects into direct, indirect, and total effects shows that the core explanatory variable—the sports industry—has a significantly positive direct effect and a significantly negative indirect effect. These findings reinforce the conclusion that the sports industry enhances economic development locally, but simultaneously produces adverse spillover effects that restrain economic growth in surrounding areas.

The negative indirect effect can be attributed to the “siphon effect” and interregional resource competition (36). The rapid development of the sports industry heavily depends on the concentrated allocation of key factors, such as capital, professional talent, and policy support. This concentration naturally attracts high-quality resources away from neighboring areas. For instance, existing literature indicates that the spatial agglomeration of head sports enterprises and the hosting of mega sporting events draw massive investments and elite talent into core cities. Consequently, this resource drain weakens the development potential of adjacent regions, generating a negative spatial spillover.

Despite these negative spillovers, the absolute value of the indirect effect is smaller than that of the direct effect, resulting in a net positive total effect. This occurs because the negative spillovers do not completely offset the robust local economic gains. Several factors explain this dynamic. First, interregional industrial complementarity plays a crucial role. While core regions dominate high-end sports services and major event hosting, neighboring areas often absorb overflow demand by undertaking complementary roles, such as basic sporting goods manufacturing or peripheral sports tourism (37). Second, macro-level policy coordination partially mitigates vicious competition, ensuring an overall expansion of the regional sports market. Thus, the net macroeconomic impact remains positive.

Overall, this pattern of “local promotion and external suppression” highlights the need for stronger interregional coordination and industrial synergy. It is important to note that the specific transmission mechanisms driving this siphon effect—such as precise capital flow trajectories and talent migration patterns—are not empirically tested in this study. Future research should construct intermediary effect models to quantitatively examine these underlying mechanisms.

## 5 Results

To examine the impact of the sports industry on regional economic development, this study employs fixed-effects models and the spatial Durbin model (SDM) using panel data from 210 province-year observations in China from 2015 to 2024. The analysis assesses the effect of the sports industry on regional economic growth and explores its underlying mechanisms. The main findings are summarized as follows.

First, the sports industry exerts a highly significant positive effect on regional economic development, indicating that the growth of the sports industry effectively promotes regional economic expansion.

Second, the impact exhibits clear regional heterogeneity: it is significant in the eastern region but not statistically significant in the central and western regions;

Third, from 2015 to 2024, the two systems show similar spatiotemporal patterns, characterized by low-value clustering and high-value polarization in eastern regions;

Fourth, spatial autocorrelation tests show that the effects are not evenly distributed but exhibit high–high and low–low clustering, indicating positive spatial autocorrelation;

Fifth, Spatial Durbin model results show that, after controlling for spatial factors, the sports industry still has a significant positive effect on regional economic development; spatial effect decomposition further shows a siphoning effect, where the development of the sports industry in one region inhibits economic development in neighboring regions.

## 6 Discussion

Empirical evidence based on China's provincial panel data from 2015 to 2024 shows that the development of the sports industry significantly promotes regional economic growth. This conclusion remains robust across different model specifications and robustness checks, thereby supporting research hypothesis H1. The results provide empirical support for the core propositions of industrial economics and endogenous growth theory, which emphasize that emerging service industries can serve as important drivers of economic growth. They also suggest that the sports industry has gradually evolved from a “marginal consumer industry” into a key engine of regional economic development (38). Unlike traditional growth models driven by investment expansion or resource dependence, the sports industry mainly affects economic performance through channels such as consumption expansion, human capital accumulation, and institutional innovation. As a result, its growth effects tend to be more sustainable and endogenous, which aligns with the “demand pull–supply upgrading–efficiency enhancement” transmission mechanism highlighted in relevant theories (28).

The regional heterogeneity analysis further indicates that the growth-enhancing effect of the sports industry is most pronounced in eastern China, whereas the effects in central and western regions are statistically insignificant and even show mildly inhibitory tendencies. This does not necessarily imply that the sports industry is “ineffective” in the central and western regions; rather, it likely reflects a mismatch between the stage of industrial development and the regional economic foundation. According to stage theory in developmental economics, when market size, consumer purchasing power, and supporting industrial systems remain relatively weak, the sports industry may face a longer investment payback period. In such contexts, the short-term growth effects may be offset by the costs associated with infrastructure construction and industrial cultivation (39). In contrast, eastern regions benefit from more mature market mechanisms, stronger consumption upgrading, and deeper integration with related sectors such as the digital economy and cultural tourism. These advantages allow the sports industry to more readily realize economies of scale and agglomeration effects, thereby amplifying its contribution to regional economic development (31). This finding extends existing conclusions that “the sports industry generally promotes economic growth” by highlighting the important moderating role of regional development conditions.

From the perspective of spatial spillover effects, regional economic development exhibits significant positive spatial autocorrelation, indicating that economic growth is not geographically isolated but instead demonstrates clear patterns of spatial agglomeration and diffusion. The spatial Durbin model results further reveal that although the sports industry promotes economic growth within a region, it generates significant negative spatial spillover effects on neighboring regions. This finding partially supports hypothesis H2b while also presenting a more complex outcome than the conventional expectation of “positive spillovers.” Drawing on new economic geography and spatial competition theory, such negative spillovers may arise from resource concentration and a “siphoning effect” in sports industry development: core regions gain advantages in event resources, capital inflows, and high-end talent allocation, which attracts production factors from surrounding areas and weakens the development potential of neighboring regions (40). Moreover, intensified interregional competition for hosting sports events, industrial layout, and consumer market contend for may further crowd out sports-related industries and associated economic activities in adjacent areas.

Importantly, this pattern of “local promotion and neighboring suppression” is not unique to the sports industry, but rather reflects a broader phenomenon commonly observed in emerging industries under conditions of uneven regional development (41). It highlights deeper issues such as incomplete regional coordination mechanisms and the need to optimize cross-regional industrial division and cooperation systems (42). Therefore, the findings not only deepen the understanding of the spatial externalities associated with sports industry development but also imply that high-quality growth of the sports industry should place greater emphasis on regional coordination and the establishment of mechanisms that facilitate rational factor mobility.

Importantly, the observed “local promotion and neighboring suppression” pattern highlights the urgent need to shift from isolated local expansion to synergistic spatial governance. To translate these findings into practical policy, it is essential to implement region-specific strategies and cross-regional coordination mechanisms tailored to China's heterogeneous realities:

First, Eastern Region: Act as a radiating growth pole by transferring capital and advanced expertise to central and western areas through innovative models like the “enclave economy” and cross-regional sports industrial parks; Second, Central Region: Focus on cultivating localized, high-quality sports event Intellectual Properties (IPs) and structurally upgrading regional sports consumption; Third,Western Region: Prioritize characteristic sports tourism and outdoor recreation based on unique ecological endowments, avoiding homogeneous competition with the more developed eastern markets; Fourth, Cross-Regional Coordination: Establish an inter-provincial joint-conference system to coordinate the bidding and spatial layout of large-scale sporting events, thereby mitigating vicious inter-regional competition and optimizing resource allocation.

Overall, this study contributes to the literature by enriching the spatial analytical perspective on the economic effects of the sports industry. From a practical standpoint, it suggests that while the sports industry serves as an important engine for regional economic growth, it may also intensify regional disparities in the absence of effective coordination mechanisms. Consequently, how to strengthen the local growth effects of the sports industry while mitigating its negative spillover impacts represents a key issue for future policymaking and further research. These findings also provide useful insights for extending related studies from the perspectives of industrial synergy, spatial governance, and institutional design.

## Data Availability

The datasets presented in this study can be found in online repositories. The names of the repository/repositories and accession number(s) can be found below: https://www.stats.gov.cn/sj/ndsj/.
